# GeneBase 1.1: a tool to summarize data from NCBI gene datasets and its application to an update of human gene statistics

**DOI:** 10.1093/database/baw153

**Published:** 2016-12-26

**Authors:** Allison Piovesan, Maria Caracausi, Francesca Antonaros, Maria Chiara Pelleri, Lorenza Vitale

**Affiliations:** Department of Experimental, Diagnostic and Specialty Medicine (DIMES), Unit of Histology, Embryology and Applied Biology, University of Bologna, Via Belmeloro 8, 40126 Bologna, Italy

## Abstract

We release GeneBase 1.1, a local tool with a graphical interface useful for parsing, structuring and indexing data from the National Center for Biotechnology Information (NCBI) Gene data bank. Compared to its predecessor GeneBase (1.0), GeneBase 1.1 now allows dynamic calculation and summarization in terms of median, mean, standard deviation and total for many quantitative parameters associated with genes, gene transcripts and gene features (exons, introns, coding sequences, untranslated regions). GeneBase 1.1 thus offers the opportunity to perform analyses of the main gene structure parameters also following the search for any set of genes with the desired characteristics, allowing unique functionalities not provided by the NCBI Gene itself. In order to show the potential of our tool for local parsing, structuring and dynamic summarizing of publicly available databases for data retrieval, analysis and testing of biological hypotheses, we provide as a sample application a revised set of statistics for human nuclear genes, gene transcripts and gene features. In contrast with previous estimations strongly underestimating the length of human genes, a ‘mean’ human protein-coding gene is 67 kbp long, has eleven 309 bp long exons and ten 6355 bp long introns. Median, mean and extreme values are provided for many other features offering an updated reference source for human genome studies, data useful to set parameters for bioinformatic tools and interesting clues to the biomedical meaning of the gene features themselves.

**Database URL**: http://apollo11.isto.unibo.it/software/

## Introduction

Genome browsers such as the Map Viewer at the National Center for Biotechnology Information (NCBI) (1), Ensembl (2) at the European Molecular Biology Laboratory-European Bioinformatics Institute (EMBL-EBI) and Wellcome Trust Sanger Institute and the University of California Santa Cruz (UCSC) Genome Browser (3) provide plenty of data about the human genome and genes, also downloadable in different types of text formats. However, they do not offer searches like the ones made possible by a fully structured database (4). In particular, numerical values for many features are not treated as database number fields, and summarization of the values in terms of mean, standard deviation (SD) and so on is often not available. When these statistics are provided (e.g. https://www.ncbi.nlm.nih.gov/genome/annotation_euk/Homo_sapiens/108/), they are based on fixed gene sets and cannot be dynamically created following searching for a set of genes with the desired characteristics (e.g. gene function or chromosomal location). Furthermore, the reported values (e.g. the length of the shortest gene) cannot be associated with the records of the specific genes having those values. Finally, different data tables presented as web pages for gene features and gene sequences are not related and cross-table searches in this sense are not possible (e.g. searching for introns ending with the CAGCAG sequence to readily identify possible candidate genes for subtle alternative splicing (5)).

To address these issues we improved GeneBase (1.0), a user-friendly local tool with a graphical interface incorporating data available in the NCBI Gene database and allowing users to perform original searches, calculations and analyses of the main gene-associated meta-information (6). The original GeneBase (1.0) has been revised adding in particular calculation and related fields leading to the release GeneBase 1.1 presented here which now allows summarization in terms of median, mean, SD and total for many quantitative parameters associated with genes, gene transcripts and gene features, dynamically calculated for any desired subset thus providing unique functionalities not offered by the NCBI Gene itself.

To demonstrate the utility of GeneBase 1.1 for understanding statistics about a whole set of genes, we provide an updated reference set of statistics of human nuclear gene parameters useful for human genome studies. Although we do not undervalue the relevance of repetitive DNA sequences which are estimated to account for 66–69% of the human genome (7), we will especially focus on sequences annotated as genes analysing data available in the NCBI Gene (1) database following parsing by GeneBase 1.1. The tool also allowed the selection of a curated subset of human nuclear genes with a ‘REVIEWED’ or ‘VALIDATED’ Reference Sequence (RefSeq) status (8). The NCBI Gene database has arbitrarily been chosen as a reference data set because being part of an international effort, it represents data that is mostly presented also by other genome browsers such as Ensembl (2) and the UCSC Genome Browser (3) which are now based mainly on GENCODE (9). Due to the high concordance of GENCODE Basic set (Only full-length, protein-coding transcripts at protein-coding genes) with NCBI RefSeq database data as proved by Frankish and coll. (10), it may derived that NCBI Gene may soundly be suitable for our purpose, although use of the other genome browsers might be a useful addition to the analysis of gene data.

In particular, here we show that substantial changes have modified these main statistics still typically provided in the literature as reference data. Numerical and statistical data related to the human genome are typically mentioned as general knowledge without citing a proper updated reference (11, 12) or from genome browser websites (13, 14). This is also due to a lack of a systematic reanalysis of these values which date back to February 2001 (15–17) in the recent literature, while the reanalysis of data provided by regularly updated genome browser websites is hampered by limitations presented above.

While showing potential of our tool for local parsing, structuring and dynamic summarizing of publicly available databases for data retrieval and analysis, we provide as a sample application a revised set of statistics for human nuclear genes which offers both an updated reference data set for human genome studies and interesting clues to the biomedical meaning of the gene features themselves.

## Methods

### Tool development

The original GeneBase (1.0) is a structured local database consisting of three main related tables (‘Gene_Summary’, ‘Gene_Table’ and ‘Gene_Ontology’) containing information such as gene nomenclature, structure and transcripts parsed from a desired NCBI Gene dataset through an initial Python script.

First, the original available Python executable script has been improved (http://www.python.org/, version 2.7) in order to obtain a ‘Gene_Table’ record also for genes lacking transcribed products but with a given genomic location, leading to the possibility to calculate, e.g. gene size of RNA transfers (tRNAs), that would have otherwise been impossible. The other script parsing functions remain unchanged, including the obtainment of the three tab-delimited files which then need to be loaded into GeneBase as provided, following the software documentation.

GeneBase 1.1 has been improved here compared with the original version especially in order to calculate specific gene and transcript feature statistics. In particular, fields related with other GeneBase 1.1 tables were added in the ‘Gene_Summary’ table in order to improve search opportunities showing, e.g. the length, the annotated strand and the transcript RefSeq status associated with each gene. A script automatically executed after the first import step was implemented in order to also calculate mature messenger RNA (mRNA), 5′ and 3′ UTR (untranslated region) lengths in the ‘Gene_Table’ table. This was not a trivial task due to the necessity to take the intron lengths that can be different for each isoform into account in the calculation. ‘Transcripts’ GeneBase 1.1 table has been expanded to also include mature transcript, CDS (coding DNA sequence), 5′ and 3′ UTR lengths and exon and coding exon number per transcript. ‘Genes’ table has been created in order to show an informational overview regarding gene lengths, transcript number for each gene and exon and coding exon number for the transcript isoform with the highest number of exons of each gene.

Furthermore, in ‘Transcripts’ and ‘Genes’ tables summary sections have been created in order to collect and calculate median, mean, SD and total values for all the available gene features. The summary section of the ‘Reports’ table, generated in the original GeneBase (1.0) version to easily provide mean exon and intron lengths, has been improved here by adding the median and the total length calculations for the current records shown in ‘Gene_Table’. In particular, all the summary sections available in GeneBase 1.1 update the listed values depending on the current found record subset, thus statistics can be dynamically calculated for any desired subset of genes.

GeneBase 1.1 was originally developed and improved here within the FileMaker Pro Advanced environment (FileMaker, Santa Clara, CA), a database management system with an intuitive user-friendly graphical interface for both Macintosh (Mac OS X) and Windows operating systems. Information has been fragmented into distinct fields as much as possible in order to facilitate independent data management. The normalization through relationships between the tables has been only partially realized in order to balance the elimination of redundancy and the speed of searches. Numbers are stored as numeric values allowing for instance record sorting by ascending or descending order and searches by range. Further data not present in the original NCBI Gene entries and extracted here from the available information is highlighted in red text. All data fields are indexed to ensure efficient data retrieval through the query options.

As for the original version, we do not provide a GeneBase 1.1 web tool because the FileMaker Pro environment does not support the full features available in the local file when the file is published via a HTML (HyperText Markup Language) interface.

### Human database construction

Improvement brought in GeneBase 1.1 makes it easier to extract statistical summary information about genes available in NCBI Gene. As a sample application of GeneBase 1.1, we provide a revised snapshot of statistics for human nuclear genes, making a stand-alone version of GeneBase 1.1 pre-loaded with available human gene data updated to January 2016 (GeneBase 1.1 Human). Although it will not be automatically kept up-to-date as other on-line gene browsers, we provide an empty template which may be used at any time to load *ab initio* the latest version or any desired subset of NCBI Gene data for any organism and by any user, following parsing by our scripts. In contrast with the first presentation of GeneBase (6), we decided to initially include in our study also gene models, in order to obtain a complete picture of human nuclear genes in the NCBI database. All the currently (‘alive’/‘live’ qualification) available human gene entries were downloaded from NCBI Gene on 19 January 2016, using the following text query: ‘Homo sapiens’[Organism] AND ‘source_genomic’[properties] AND alive[property].

In order to integrate exon and intron nucleotide sequences, all the human chromosome sequences were downloaded from the NCBI Nucleotide database using Batch Entrez (http://www.ncbi.nlm.nih.gov/sites/batchentrez) in FASTA format on 26 January 2016 (corresponding to GRCh38.p2, incorporating centromere models), parsed using the available Python executable script and imported into GeneBase 1.1 Human, thoroughly following the software documentation. Sequences are not required to calculate statistical summary information, thus if the user is interested only in gene features such as gene number and size statistics, this step is not necessary.

We made Mac OS X and Windows stand-alone software (of both GeneBase 1.1 pre-loaded and empty versions), including the FileMaker runtime with a user guide provided and the relative Python scripts for the initial data pre-processing and sequence calculations, freely available to all for basic use at http://apollo11.isto.unibo.it/software/. The freely distributed licensed runtime application allows full data import, records export in diverse file formats, as well as full record management and analysis and script execution. The downloading, parsing and import of gene entries, the downloading of chromosome sequences and the calculation of exon and intron sequences and the improvements are described in detail in the software documentation. An original copy of FileMaker Pro version 12 (or higher) is required only for the modification of the tool for personal purposes (creation of new fields, further calculation or additional relationship definition).

### Sample application: statistics of the human gene features

In order to provide a revised set of statistics regarding human nuclear genes and transcripts through GeneBase 1.1 Human, we considered only genes with ‘REVIEWED’ or ‘VALIDATED’ RefSeq status, with at least one ‘REVIEWED’ or ‘VALIDATED’ transcript, excluding ‘not in current annotation release’ records (‘Genome_Annotation_Status’ field). This selection has already been proven successful in excluding from calculations model RefSeq records generated by automated pipelines (6). As mentioned above, GeneBase has been improved here and the fields useful for this gene selection are available in each software table. In particular, the exon and intron non-redundant sets were found counting only one exon or intron for each group of exons or introns present in multiple transcript isoforms. Specific GeneBase 1.1 searches performed to find numbers cited in Figures, Tables, Supplementary data and throughout the text are detailed in the Supplementary Methods file. The Supplementary Methods file also describes the standard ‘Find’ and ‘Sort’ FileMaker Pro functions which allow searching and sorting by any column in each GeneBase 1.1 table (please see the user guide provided at http://apollo11.isto.unibo.it/software/ for further details).

## Results

### Database functionality

GeneBase 1.1 is now composed of six related tables: ‘Gene_Summary’, ‘Gene_Table’, ‘Gene_Ontology’, ‘Reports’, ‘Trascripts’ and ‘Genes’. The first three tables include information which needs to be extracted and parsed from NCBI Gene entries (please see the user guide provided at http://apollo11.isto.unibo.it/software/ for further details), while ‘Reports’, ‘Transcripts’ and ‘Genes’ tables provide an overview of the main available gene and transcript features and summary sections created in order to collect and calculate their median, mean, SD and total values. In particular, a statistical summary for exon and intron lengths is available in the ‘Reports’ table. ‘Transcripts’ table provides statistical values for transcript length, CDS, 5′ and 3′ UTR lengths and exon and coding exon number per transcript. From ‘Genes’ table, it is possible to retrieve statistical values for the gene length, the number of transcripts per gene and the number of exons and coding exons for the longest transcript associated with each gene.

The most relevant functionality introduced in GeneBase 1.1 is that these statistics can be dynamically calculated for any desired subset of genes, making it easier to extract summary values for the subset of ‘REVIEWED’ and ‘VALIDATED’ human nuclear genes, as described below.

We obtained 59 801 entries from downloading all current live human records with a genomic gene source (Methods) from NCBI Gene available up to 19 January 2016.

Following the initial parsing and importing steps (Methods), the three main tables in GeneBase 1.1 Human database are constituted as follows: ‘Gene_Summary’ contains 59 801 records (one for each NCBI Gene entry). ‘Gene_Table’ contains 1 502 237 records (one record for each gene exon, including the downstream intron if an intron follows that exon), corresponding to 40 942 genes with 136 694 transcripts in total (equal to the ‘Transcripts’ table record number), excluding genes without annotated transcribed products. ‘Gene_Ontology’ contains 18 726 records in all, one for each gene with Gene Ontology information available.

**Figure 1.**
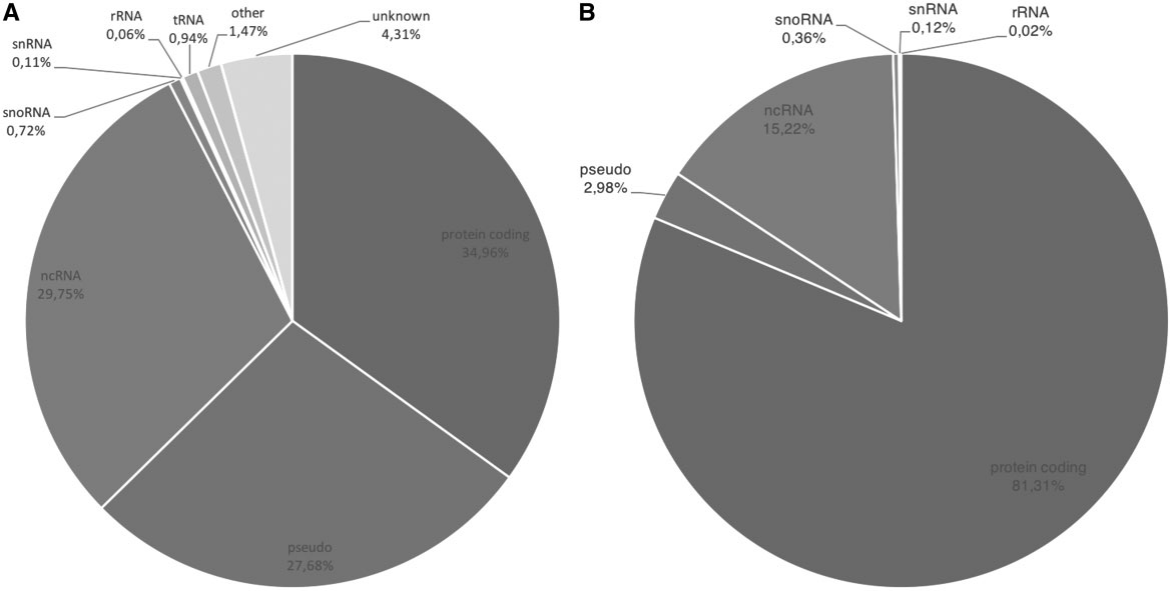
(A) Gene type composition of GeneBase 1.1 Human entries for a total of 59 801 genes and (B) for 22 451 ‘REVIEWED’ or ‘VALIDATED’ genes with at least one ‘REVIEWED’ or ‘VALIDATED’ transcript (genes not in current annotation release are excluded). Gene type labels are derived from ‘Gene_Type’ field of GeneBase 1.1 Human ‘Gene_Summary’ table as annotated in NCBI Gene as follows: protein-coding, pseudo (pseudogenes), ncRNA (non-coding RNA), snoRNA (small nucleolar RNA), snRNA (small nuclear RNA), rRNA (ribosomal RNA), tRNA (transfer RNA), ‘other’ and ‘unknown’.

The overall gene type composition of GeneBase 1.1 Human is shown in Figure 1A (including ‘REVIEWED’, ‘VALIDATED’, ‘PROVISIONAL’, ‘PREDICTED’, ‘INFE RRED’ and ‘MODEL’ RefSeq status entries); Figure 1B is the representation of 22 451 GeneBase 1.1 Human gene entries with ‘REVIEWED’ or ‘VALIDATED’ RefSeq status, with at least one ‘REVIEWED’ or ‘VALIDATED’ transcript and excluding genes not in current annotation release, corresponding to a total of 45 541 transcripts, which is the subset that will be considered onwards. The available RefSeq statuses associated with genes and the corresponding RNAs are summarized in Supplementary Table S1; it should be noted that the RefSeq status of a particular RNA may be different from the status assigned to the corresponding gene.

In order to integrate exon and intron nucleotide sequences, only entries with the ‘REVIEWED’ or the ‘VALIDATED’ RefSeq status having an ‘NM_’ or ‘NR_’ type of RefSeq RNA accession number (in order to exclude ‘XM_’ or ‘XR_’ model Refseq records generated by automated pipelines) were selected. After the chromosome sequence download, parsing and importing steps (Methods), a total of 459 868 ‘Gene_Table’ records were updated with exon, coding exon (for protein-coding transcript isoforms) and the corresponding downstream intron sequences up to 26 January 2016. The whole database including sequences has a size of 6.43 gigabytes following decompression.

### Revision of human nuclear gene feature statistics

**Figure 2.**
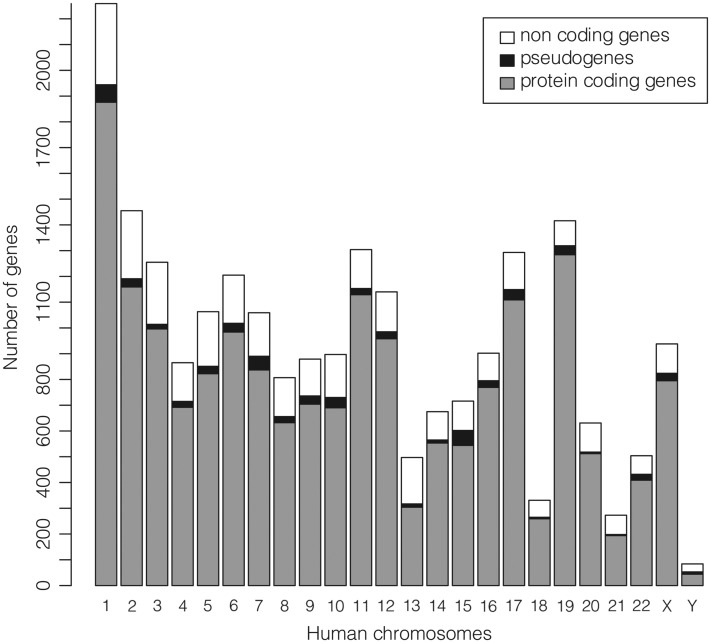
Number of ‘REVIEWED’ or ‘VALIDATED’ genes with at least one ‘REVIEWED’ or ‘VALIDATED’ transcript in GeneBase 1.1 Human (genes not in current annotation release are excluded) divided in protein-coding genes, pseudogenes and non-coding genes (which include genes for ribosomal RNAs, small nucleolar RNAs, small nuclear RNAs and non-coding RNAs) for each human chromosome. See Table 1 and Supplementary Table S2 for more details.

The subset of the 22 451 ‘REVIEWED’ or ‘VALIDATED’ gene entries with at least one ‘REVIEWED’ or ‘VALIDATED’ transcript (excluding genes not in current annotation release) available in GeneBase 1.1 Human for each human chromosome is shown in Figure 2 (Table 1) and includes a total of 18 255 protein-coding genes, 668 pseudogenes and 3528 non-coding genes (Supplementary Table S2). The location of the 50.42% of genes has the transcript sequence on the DNA strand annotated as ‘plus’.

**Table 1.** Human chromosome lengths and number of genes

Chromosome	Length (Mb)^a^	Number of genes per chromosome in NCBI Genome^a^	Number of genes per chromosome in GeneBase 1.1 without selection	Number of ‘REVIEWED’ and ‘VALIDATED’ genes per chromosome in GeneBase 1.1 Human^b^
1	**248.96**	**9823**	**5523**	**2259**
2	242.19	7746	4215	1455
3	198.30	5855	3287	1255
4	190.21	4905	2649	865
5	181.54	5006	2795	1063
6	170.81	5843	3364	1205
7	159.35	5426	3010	1059
8	145.14	4292	2383	807
9	138.39	4670	2509	879
10	133.80	4385	2379	897
11	135.09	5571	3235	1304
12	133.28	4942	2731	1140
13	114.36	2880	1537	497
14	107.04	3965	2220	675
15	101.99	3791	2081	716
16	90.34	3763	2206	902
17	83.26	4686	2694	1293
18	80.37	2062	1118	331
19	58.62	4480	2694	1416
20	64.44	2600	1435	631
21	**46.71**	1526	834	273
22	50.82	2314	1356	504
X	156.04	3690	2416	938
Y	57.23	**1092**	**574**	**84**
Total	3088.27	50 704	59 245^c^	22 448^d^

Mb: megabase.

a These columns shows numbers reported at http://www.ncbi.nlm.nih.gov/genome accessed on 19 January 2016 (when NCBI Gene entries were also downloaded for parsing and import in GeneBase 1.1).

b This column shows the number of gene entries with ‘REVIEWED’ or ‘VALIDATED’ RefSeq status, with at least one ‘REVIEWED’ or ‘VALIDATED’ transcript, excluding ‘not in current annotation release’ records in the ‘Gene_Summary’ table of GeneBase (Methods; Supplementary Methods File).

c The three remaining genes (to reach the total of 59 801 genes) are 65 genes with unknown and 491 empty ‘Chromosome’ field.

d The three remaining genes (to reach the total of 22 451 genes) are three pseudogenes with unknown location (Gene IDs: 100233156, 283788, 389834).

Bold: minimum and maximum values for each column. Underlined: the three smallest numbers of genes per chromosome, beyond chrY.

See Supplementary Table S2 for more details on gene types.

**Figure 3.**
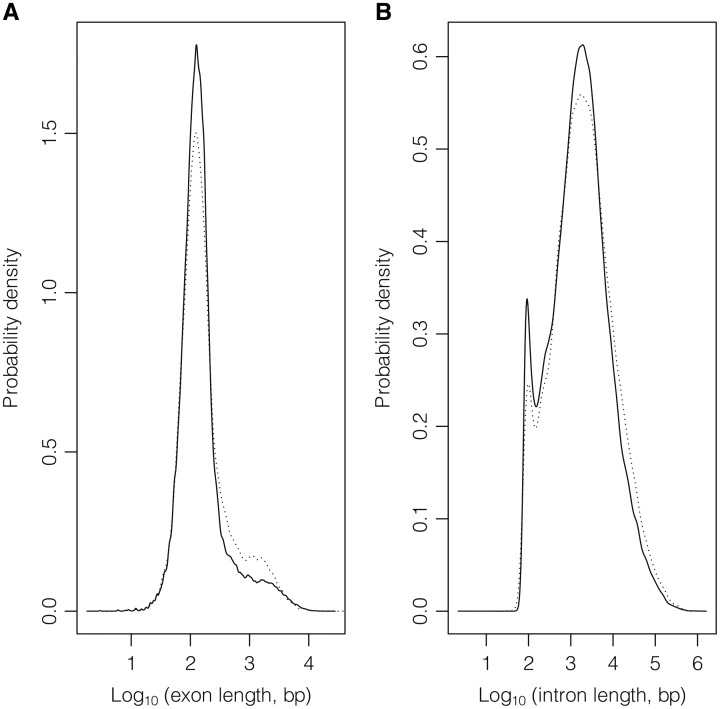
Exon (A) and intron (B) length distributions considering GeneBase 1.1 Human ‘Gene_Table’ records with a ‘VALIDATED’ or ‘REVIEWED’ RefSeq status, with an ‘NM_’ (protein-coding RNAs, continuous lines) or ‘NR_’ (non-coding RNAs, dotted lines) type of corresponding RefSeq RNA accession number, belonging to ‘REVIEWED’ or ‘VALIDATED’ genes excluding those not in current annotation release.

In the considered subset of ‘REVIEWED’ and ‘VALIDATED’ entries, Table 2 shows statistics about number and length of both protein-coding and non-coding genes; transcript (Supplementary Figure S1), exon (Figure 3A, Supplementary Figure S2) and intron (Figure 3B) data are provided in Tables 3 and 4. Supplementary Table S3 gives these statistics for protein-coding and non-coding genes counted together.

**Table 2.** Known human nuclear gene numbers and lengths

	Protein-coding genes	Non-coding genes
Total number of entries^a^	18 255	4196
Median number per chr	737 per chr	175 per chr
Mean number per chr	761 per chr	175 per chr
SD	400 per chr	75 per chr
Minimum number	44 (chrY)	40 (chrY)
Maximum number	1876 (chr 1)	383 (chr1)
Median length	26 288 bp	11 155 bp
Mean length	66 577 bp	33 803 bp
SD	130 398 bp	68 302 bp
Shortest	189 bp (*KRTAP6-2*, chr21)	60 bp (*MIR4722*, chr16)
Longest	2 473 559 bp (*RBFOX1*, chr16)	1 033 350 bp (*PTCHD1-AS*, chrX)
Total length	1 215 363 666 bp	141 838 888 bp

SD: standard deviation; chr: chromosome; bp: base pair.

a We considered only protein-coding or non-coding genes with ‘REVIEWED’ or ‘VALIDATED’ RefSeq status, with at least one ‘REVIEWED’ or ‘VALIDATED’ transcript, excluding ‘not in current annotation release’ records in the ‘Gene_Summary’ and ‘Genes’ tables of GeneBase 1.1 Human as explained in the ‘Methods’ section and in the Supplementary Methods File.

The mean, minimum and maximum gene numbers are also available in Supplementary Table S2.

**Table 3.** Human protein-coding transcript (mRNA), exon and intron numbers and lengths

		mRNAs	Exons	Coding Exons^b^	Introns
Number	Total of entries^a^	37 608	412 641	384 289	375 033
			non-redundant: 147 484	non-redundant: 138 736	non-redundant: 134 497
	Median	4.0	8.0	8.0	7.0
		per gene	per transcript	per transcript	per transcript
	Mean	5.4	11.0	10.2	10.0
		per gene	per transcript	per transcript	per transcript
	SD	5.4	9.9	9.9	8.9
		per gene	per transcript	per transcript	per transcript
	Min	1	1	1	1
		(3984 genes)	(948 transcripts, 942 genes)	(2560 transcripts, 1856 genes)	(1724 transcripts, 1441 genes)
	Max	28	363	362	362
		(*CACNA1G*, chr17)	(*TTN*, chr2)	(*TTN*, chr2)	(*TTN*, chr2)
Length	Median	2787 bp	133 bp non-redundant: 141 bp	122 bp	1,632 bp
			126 bp^c^ non-redundant: 130 bp^c^	non-redundant: 122 bp	non-redundant: 1710 bp
	Mean	3392 bp	309 bp non-redundant: 365 bp	163 bp	6,355 bp
			161 bp^c^ non-redundant: 175 bp^c^	non-redundant: 171 bp	non-redundant: 6990 bp
	SD	2600 bp	725 bp non-redundant: 810 bp	256 bp	20 649 bp
			214 bp^c^ non-redundant: 240 bp^c^	non-redundant: 290 bp	non-redundant: 23 493 bp
	Shortest^d^	186 bp	2 bp	1 bp	30 bp
		(*DEFB133*, chr6)	(*SEPT7*, exon 2)	(*GSTP1*, last base of exon 1)	(*MST1L*, intron 9)
	Longest	109 224 bp	24 927 bp	21 693 bp	1 160 411 bp
		(*TTN*, chr2)	(*ZBTB20*, exon 5, last, with 422 coding bp only)	(*MUC16*, exon 3)	(*ROBO2*, intron 2)
	Total	127 583 379 bp	127 583 379 bp	62 554 408 bp	2 383 497 318 bp
			non-redundant: 53 827 863 bp	non-redundant: 23 698 355 bp	non-redundant: 940 173 183 bp

SD: standard deviation; min: minimum; max: maximum; chr: chromosome; bp: base pair.

a We considered only protein-coding genes with ‘REVIEWED’ or ‘VALIDATED’ RefSeq status, with at least one ‘REVIEWED’ or ‘VALIDATED’ transcript, excluding ‘not in current annotation release’ records in GeneBase 1.1 Human software. Non-coding RNA produced by a protein-coding gene are excluded, selecting only transcripts with an ‘NM_’ RNA accession number type (Methods and Supplementary Methods File). In particular, mRNA data were derived from the ‘Genes’ and ‘Transcripts’ tables, while exon and intron data from ‘Gene_Table’ and ‘Reports’ tables. Exon and intron non-redundant sets were found counting only one exon or intron for each group of exons or introns present in multiple transcript isoforms. A comprehensive analysis including both non-coding and protein-coding genes is available in the Supplementary Table S3.

b In this column, numbers and lengths are shown considering only the protein-coding portion of exons, including stop codons.

c These values were calculated excluding 37 608 records corresponding to the last exon, which is usually the longest one (Supplementary Figure S2). (Values considering last exons only: 1264 bp median, 1782 bp mean, 1709 SD. Values considering non-redundant last exons only: 1177 bp median, 1716 bp mean, 1683 bp SD).

d Exon and intron minimum lengths are adjusted following manual curation as described (6) because the starting database contains some artefactual data.

**Table 4.** Human non-coding RNA, exon and intron numbers and lengths

		Non-coding RNAs	Exons	Introns
Number	Total of entries^a^	7933	47 521	39588
			non-redundant: 24 783	non-redundant: 21 297
	Median	1 per gene	4.0	3.0
			per transcript	per transcript
	Mean	1.3 per gene	6.0	5.0
			per transcript	per transcript
	SD	0.9 per gene	5.8 per transcript	4.8 per transcript
	Min	1 (4728 genes)	1	1
			(554 transcripts and genes)	(908 transcripts, 792 genes)
	Max	52 (*UTY*, chrY)	51	50
			(*LOC100499484-C9ORF174*, chr9)	(*LOC100499484-C9ORF174*, chr9)
Length	Median	1787 bp	141 bp non-redundant: 153 bp	1855 bp
			125 bp^b^non-redundant: 130 bp^b^	non-redundant: 2089 bp
	Mean	2168 bp	362 bp non-redundant: 405 bp	7897 bp
			177 bp^a^ non-redundant: 199 bp^a^	non-redundant: 9873 bp
	SD	2037 bp	863 bp non-redundant: 979 bp	22 390 bp
			237 bp^b^ non-redundant: 296 bp^b^	non-redundant: 26 807 bp
	Shortest	60 bp	4 bp	31 bp
		(*MIR4722*, chr16)	(*MT1IP*, exon 2)	(*UCKL1-AS1*, unique intron)
	Longest	91 671 bp	91 671 bp	499 303 bp
		(*KCNQ1OT1*, chr11)	(*KCNQ1OT1*, unique exon)	(*C8orf37-AS1*, intron 2)
	Total	17 200 281 bp	17 200 281 bp	312 610 461 bp
			non-redundant: 10 040 755 bp	non-redundant: 210 269 817 bp

SD: standard deviation; min: minimum; max: maximum; chr: chromosome; bp: base pair.

a We considered only genes with ‘REVIEWED’ or ‘VALIDATED’ RefSeq status, with at least one ‘REVIEWED’ or ‘VALIDATED’ transcript, excluding ‘not in current annotation release’ records in GeneBase 1.1 Human software. Here only transcripts with an ‘NR_’ RNA accession number type (Methods and Supplementary Methods File) are selected, corresponding to 5890 genes (since also non-coding RNAs can be transcribed from a protein-coding gene). In particular, transcript data were derived from the ‘Genes’ and ‘Transcripts’ tables, while exon and intron data were derived from ‘Gene_Table’ and ‘Reports’ tables. Exon and intron non-redundant sets were found counting only one exon or intron for each group of exons or introns present in multiple transcript isoforms. A comprehensive analysis including both non-coding and protein-coding genes is available in the Supplementary Table S3.

b These values were calculated excluding 7933 records corresponding to the last exon, which is usually the longest one (Supplementary Figure S2). (Values considering non-redundant last exons only: 821 bp median, 1287 bp mean, 1775 SD. Values considering non-redundant last exons only: 676 bp median, 1161 bp mean, 1851 bp SD).

It can be noted that the 66.56% of considered genes (14 944 out of the total of 22 451 genes) transcribes for at least two transcript isoforms. Regarding splicing, out of a total of 414 385 available intron sequences, the overriding majority (98.95%, 410 038 introns) presents canonical splice donor and acceptor sites (GT and AG, respectively); 0.87% (3,594 introns) are of the GC–AG type and 0.11% (439 introns) AT–AC; among the remaining, the majority uses at one boundary one canonical splice site (GT or AG) in combination with a non-canonical one at the other boundary (Supplementary Table S4).

Data on the length of the specific mRNA regions are available in Table 5. The main known non-coding RNA types are in Supplementary Table S5.

**Table 5.** Human mRNA region numbers and lengths

	5′ UTR	CDS	3′ UTR
Median length	203 bp	1278 bp	938 bp
Mean length	259 bp	1663 bp	1470 bp
SD	228 bp	1901 bp	1620 bp
Shortest	0 bp	75 bp (*MTRNR2L1*, chr17)	0 bp
Longest	14705 bp (*SAYSD1*, chr6)	107 976 bp (*TTN*, chr2)	24 505 bp (*ZBTB20*, chr3)
Total length	9 740 061 bp	62 554 408 bp	55 288 737 bp

SD: standard deviation; UTR: untranslated region; CDS: coding DNA sequence; bp: base pair; chr: chromosome.

We considered only genes with ‘REVIEWED’ or ‘VALIDATED’ RefSeq status, with at least one ‘REVIEWED’ or ‘VALIDATED’ transcript, excluding ‘not in current annotation release’ records in GeneBase 1.1 Human software. In particular, data were derived from the ‘Transcripts’ table. Here only transcripts with an ‘NM_’ RNA accession number type (Methods and Supplementary Methods File) are selected. 5′ and 3′ UTRs minimum lengths are subjected to the quality of the RefSeq annotation.

About 39.35% of the sequences of nuclear DNA correspond to genes coding for proteins. In our data set, only 4.59% of the sequences of nuclear DNA correspond to non-coding genes. Protein-coding and non-coding genes together account for the 43.95% of chromosome sequences. The remaining part of the genomic DNA, approximately 1/2, seems to be extragenic DNA or intergenic, since it does not contain canonical genes and is interposed between them.

In addition, considering the non-redundant set of exons (without accounting for the occurrence of an exon more times in different transcript isoforms), on average, only 4.43% of the DNA sequence of a gene is part of a mature mRNA which is constituted by the sum of exons only; exons thus correspond to 1.74% of the total genome. Since, in turn, only 44.03% of the exon sequences is encoding *stricto sensu*, i.e. consists of triplets of bases which can be effectively translated into a sequence of amino acids, it can be deduced that <1% (0.77%) of the genome is coding in the strict sense, which corresponds to a total of 23.7 Mbp if we consider the non-redundant set of coding exons (counting only one coding exon for each group of exons present in multiple transcript isoforms). The remaining fraction of the nuclear DNA gene sequence is mainly composed of introns, transcribed but not translated sequences localized to the 5′ and 3′ ends of the gene (5′ and 3′ UTR) and regulatory regions.

## Discussion

We have presented here an improved, new version of our original GeneBase (1.0), a tool with a graphical interface able to parse, structure and index the whole text-based NCBI Gene content (6). Remarkably, the tool offers the possibility to manage sequences related to gene features, providing the way to integrate analysis of nucleic acid sequences and structural and functional annotation of gene sequence elements. For example, the availability of Gene Ontology is also relevant because function conservation may still be present without any sequence similarity (18).

Main changes in the new version 1.1 regard the addition of number fields useful for calculating characteristic length of the gene elements and the implementation of new tool tables useful in order to show related information about genes and transcripts, also giving the opportunity to find new relationships among their features. In particular, summary sections have been created in order to collect and calculate median, mean, SD and total values for all the available features. These values automatically update depending on the current found record subset, giving users the freedom to customize statistics which can be dynamically calculated for any desired subset of genes.

Advances implemented in the 1.1 version of GeneBase have been critical in making the use of the tool possible for the innovative purpose of generating a set of detailed statistics about a set of genes. As a sample application of GeneBase 1.1, we have provided an analysis of main statistics for annotated human nuclear genes updated to January 2016. GeneBase 1.1 has been filled with all known human nuclear genes (GeneBase 1.1 Human) as previously described (6), except for the inclusion of gene models; this decision has caused the presence in the database of a high number of genes without a transcribed product, as expected, giving on the other hand the opportunity to include genes for tRNAs.

Several results obtained by GeneBase 1.1 Human offer the possibility to obtain quantitative parameters associated with genes, gene transcripts and gene features as interesting clues to their biomedical meaning as discussed below.

The total number of human protein-coding genes and pseudogenes annotated in the NCBI Gene up to January 2016 (and thus available in GeneBase 1.1 Human, Supplementary Table S2) and in GENCODE (http://www.ensembl.org/Homo_sapiens/Info/Annotation) data sets is almost comparable (20 909 and 16 555 versus 20 313 and 14 453, respectively), while there is a greater difference between the numbers of non-coding genes (18 882 versus 25 180). In addition, considering in GeneBase 1.1 Human the subset of genes with at least one RNA having a ‘REVIEWED’ or ‘VALIDATED’ RefSeq status for our sample application trimmed 81% of non-coding genes (from 18 882 to 3528 entries retained) and 95% of pseudogenes (from 16 555 to 668 gene entries retained), while it did not affect protein-coding genes in a large measure (from 20 909 to 18 255). On one hand, this reflects the great uncertainty still surrounding non-coding genes; on the other hand, this protein-coding gene subset represents the most reliable data confirmed by the fact that this number (18 255) is very close to the last estimate (19 000) obtained through the analysis of large-scale proteomic experiments (19). Furthermore, since all these kinds of analyses depend on the chosen gene entry subset, the classification system and are subject to the accuracy of the input dataset, we decided to perform this selection in order to exclude erroneous data that were not manually verified (6, 20, 21).

It is unlikely that the data shown here could undergo significant changes when those related to genes that still remain to be characterized in detail will be included. Furthermore, we always study a consensus ideal genome in no more existing cells due to the theoretical impossibility to determine the whole sequence in living cells (22). However, human gene feature estimates differ significantly from those reported in the first preliminary article on the sequence of the human genome published in Nature (15) and have never been systematically revised since then. For example, the average size of a human protein-coding gene had been evaluated as 27 kbp (median: 14 kbp) (15), whereas now analysing the currently available data, it is of 67 kbp (median: 26 kbp, Table 2). This is likely due to a progressive increase in the genomic organization determination accuracy of the genes following the burst of expressed sequence tags (EST) database (23), in which >5.2 million of human transcript related sequences were deposited from 2001 to 2007, therefore after the publication in 2001 of the two human genome seminal reports. This subset accounts for 61% of the whole human EST database, allowing over the years the progressive merging of EST clusters mapped in the UniGene database into longer transcripts thus bridging gaps between apparently different loci. On average, a non-coding gene is half as long (34 kbp, Table 2). The absolute size, however, may vary across a range of variation of four orders of magnitude (10 000 times). Using an original method for transcriptome mapping (24), including systematic UniGene based conversion of gene identifiers (25), the estimation of the average human gene length was useful in order to determine the significance of over- or under-expressed genomic segments equivalent to single gene size in the whole normal human heart transcriptome map (26).

The largest currently known human gene, *RBFOX1* (RNA binding protein, fox-1 homolog 1), spans 2.47 Mbp on chr16 (Table 2). The other known human genes exceeding 2 Mbp in length are *CNTNAP2* (contactin associated protein-like 2, spanning 2.30 Mbp on chr7), *PTPRD* (protein tyrosine phosphatase, receptor type D, 2.30 Mbp on chr9) and *DMD* (dystrophin, 2.22 Mbp on chrX). It is reasonable to think that the length of a gene can be one of the factors that influence its probability of being interrupted when there is a chromosomal lesion or to mutate following point errors of DNA replication. Interestingly, for these genes mutations and clinical phenotypes have been described (27–29) and since their correlations with gene length do not yet appear to have been systematically studied to date, investigations in the field will be made easier by the systematic dataset we present here.

The chromosome (chr) with the smallest number of genes is chrY, followed by chr21 (which is also the shortest), chr18 and chr13. Significantly, since 1959–1960, the only three autosomal trisomies allowing live births have known to be the ones of human chromosomes 13, 18 and 21 (13). Only after 2000, we know that these chromosomes are exactly the three having the lowest number of genes in absolute in the human genome (497, 331 and 273 known genes respectively, according to GeneBase 1.1 Human database, Table 1). In these cases, despite the three not being the shortest of the human genome, the survival is progressively worsening (30) in proportion to the gene number on the chromosome suggesting that the damage derived from the general over-expression of an entire set of genes may be somewhat compensated until a certain point.

Alternative promoters, splicing and polyadenylation are the main processes leading to complex multi-transcript systems (31–33). The gene with the highest number of protein-coding transcript isoforms (10) is *CACNA1G* (calcium voltage-gated channel subunit alpha1 G, chr7, Table 3), recently associated with spinocerebellar ataxia (34). The gene with the overall highest number of described protein-coding and non-coding transcript isoforms is *UTY* (ubiquitously transcribed tetratricopeptide repeat containing, Y-linked) with at least 77 known alternative transcripts (Table 4 and Supplementary Table S3) of which the full role is still unclear (35, 36).

The average size of a human intron is 6355 bp in protein-coding genes and 7897 in non-coding genes, but in larger genes a single intron of 1 Mbp upper length (Tables 3 and 4; Supplementary Table S3) can be found. Again this estimation is substantially different from the one provided in the first genome draft of 3365 bp for protein-coding gene introns (15). The longest intron (1 160 411 bp) belongs to *ROBO2* (roundabout guidance receptor 2, chr3). Due to the presence of artefactual data in some records, manual curation is needed when considering extremely low values, as previously discussed (6). Following validation as described (6), we confirm that there are no human (and actually of any species) introns shortest than 30 bp (Table 3). About 554 protein-coding and 948 non-coding transcripts (corresponding to a total of 1496 genes) are intronless (monoexonic), representing 3.3% out of the total considered transcript set.

The average length of a human exon is only 309 bp in protein-coding genes and 362 bp in non-coding genes, but also in this case the existing variability within the human genome is very high. Notably, thanks to the GeneBase 1.1 architecture, it is possible to analyse exon lengths which vary a lot depending on whether it is a first, internal or terminal exon and whether it is coding or not (Supplementary Figure S2). For example, excluding last exons, whose sequence is mainly non-coding (because they typically contain the stop codon), the mean exon length in protein-coding genes is of 161 bp, again an estimation substantially different from the mean value provided in the first genome draft of 145 bp (for protein-coding gene internal exons). The ratio between intron and exon length (6355:309 and 7897:362 bp for protein-coding and non-coding transcripts, respectively) is about 21:1. If exon and intron lengths of protein-coding and non-coding transcripts are comparable, the factor accounting for the different gene lengths (one double of the other) is the mean number of exons, which is equal to 11 for protein-coding genes versus 6 of non-coding genes.

The average size of a human mRNA is 3392 bp (Table 3) of which only half (49.03%, Tables 3 and 5), on average, is coding. This average size much lower than that of the gene is the operative basis making the insertion of most human cDNAs (complete human DNA copy of mRNA) in many types of vectors used for gene transfer into cells possible. The mean mRNA size is included between the 28S and 18S rRNA sizes (5070 and 1869 bp, respectively, Supplementary Table S5), thus explaining the typical intensity pattern of mRNA fractioned in agarose gels with an enrichment between 28S and 18S rRNA bands. Table 5 also shows the artefactual 0 bp length of the 5′ and 3′ UTRs, thus in a certain number (203) of entries only the coding sequence has been registered. This reflects the well-known and still present difficulties in the determination of the full-length RNA (9), especially at the 5′ end (37, 38) and the characterization analyses in this field are still necessary. Notably, the extended length of 3′ UTR in comparison to 5′ UTR may have a biological explanation because it is enriched in several regulatory motifs such as adenylate uridylate (AU)-rich elements and binding sites for microRNAs, RNA-binding proteins and long non-coding RNAs (11).

The reference values for human gene elements also have a crucial key application in high throughput RNA sequencing methods (RNA-Seq), where alignment software often gives the possibility to configure custom parameter settings such as minimum and maximum intron lengths, essential for novel junction discovery (as for example in the TopHat mapper tool available in Galaxy platform, https://usegalaxy.org/). Other parameters are, e.g. mean mRNA length, mean exon length (including last exons) and average number of exons per transcript (14). Parameters of protein-coding genes that were recently estimated differently considering different databases (13), in absence of a systematic revision, include the average genomic size of genes [27 kbp in (13) versus 67 kbp here], the average number of transcripts per gene [1.4–2.7 in (13) versus 5 here], the average number of exons per transcript [7.7–10.9 in (13) versus 11 here], the average exon size [122 bp in (13) versus 161 bp here] and the total exonic genome size [78 Mbp in (13) versus 128 Mbp here]. Recent studies estimate that >85% of the genome is transcribed (39), a portion greater than the 1/2 that we have found considering only characterized sequences, pointing out again that a great effort is still needed in the annotation process for all reasons highlighted in this discussion.

In conclusion, here we present an original tool which allows parsing, structuring and dynamic summarizing data from NCBI Gene data bank. Furthermore it makes the analysis of the main gene and transcript structure parameters possible also following the search for a set of genes with the desired characteristics. In addition, we show its usefulness for a systematic revision of the main reference parameters updated to January 2016 for a description of the human nuclear gene structure. Beyond the other possible applications described here, it might be useful to design experiments for poorly characterized annotated genome regions, as in our current annotation effort of the recently defined highly restricted Down Syndrome critical region (HR-DSCR) for example, which to date does not contain known genes (40) and is fundamental to understanding genotype–phenotype relationships of Down syndrome and for identifying new therapeutic approaches (41). This analysis finally paves the way for similar studies in other organisms, possibly providing new insight on gene and genome evolution.

## Availability

GeneBase 1.1 (both pre-loaded with human nuclear gene data and empty versions), the user guide and the relative Python scripts for the initial data pre-processing and sequence calculations are publicly available at http://apollo11.isto.unibo.it/software/.
